# Probiotic paradox: bacillibactin from *Bacillus velezensis* drives pathogenic *Vibrio alginolyticus* proliferation through siderophore piracy

**DOI:** 10.1093/ismeco/ycaf132

**Published:** 2025-08-01

**Authors:** Yanhua Zeng, Haimin Chen, Xiaoxiao Gong, Manwei Jiang, Ni Liu, Wen Li, Na Zhang, Hao Long, Aiyou Huang, Zhenyu Xie

**Affiliations:** Key Laboratory of Tropical Hydrobiology and Biotechnology of Hainan Province, School of Marine Biology and Fisheries, Hainan University, Haikou, Hainan 570228, PR China; Wenchang Advanced Fisheries Research Institute, Hainan University, Wenchang, Hainan 571300, PR China; State Key Laboratory of Marine Resource Utilization in South China Sea, Hainan University, Haikou, Hainan 570228, PR China; Key Laboratory of Tropical Hydrobiology and Biotechnology of Hainan Province, School of Marine Biology and Fisheries, Hainan University, Haikou, Hainan 570228, PR China; Wenchang Advanced Fisheries Research Institute, Hainan University, Wenchang, Hainan 571300, PR China; Key Laboratory of Tropical Hydrobiology and Biotechnology of Hainan Province, School of Marine Biology and Fisheries, Hainan University, Haikou, Hainan 570228, PR China; Wenchang Advanced Fisheries Research Institute, Hainan University, Wenchang, Hainan 571300, PR China; Key Laboratory of Tropical Hydrobiology and Biotechnology of Hainan Province, School of Marine Biology and Fisheries, Hainan University, Haikou, Hainan 570228, PR China; Wenchang Advanced Fisheries Research Institute, Hainan University, Wenchang, Hainan 571300, PR China; Key Laboratory of Tropical Hydrobiology and Biotechnology of Hainan Province, School of Marine Biology and Fisheries, Hainan University, Haikou, Hainan 570228, PR China; Wenchang Advanced Fisheries Research Institute, Hainan University, Wenchang, Hainan 571300, PR China; Key Laboratory of Tropical Hydrobiology and Biotechnology of Hainan Province, School of Marine Biology and Fisheries, Hainan University, Haikou, Hainan 570228, PR China; Wenchang Advanced Fisheries Research Institute, Hainan University, Wenchang, Hainan 571300, PR China; Key Laboratory of Tropical Hydrobiology and Biotechnology of Hainan Province, School of Marine Biology and Fisheries, Hainan University, Haikou, Hainan 570228, PR China; Wenchang Advanced Fisheries Research Institute, Hainan University, Wenchang, Hainan 571300, PR China; Key Laboratory of Tropical Hydrobiology and Biotechnology of Hainan Province, School of Marine Biology and Fisheries, Hainan University, Haikou, Hainan 570228, PR China; Wenchang Advanced Fisheries Research Institute, Hainan University, Wenchang, Hainan 571300, PR China; State Key Laboratory of Marine Resource Utilization in South China Sea, Hainan University, Haikou, Hainan 570228, PR China; Key Laboratory of Tropical Hydrobiology and Biotechnology of Hainan Province, School of Marine Biology and Fisheries, Hainan University, Haikou, Hainan 570228, PR China; Wenchang Advanced Fisheries Research Institute, Hainan University, Wenchang, Hainan 571300, PR China; Key Laboratory of Tropical Hydrobiology and Biotechnology of Hainan Province, School of Marine Biology and Fisheries, Hainan University, Haikou, Hainan 570228, PR China; Wenchang Advanced Fisheries Research Institute, Hainan University, Wenchang, Hainan 571300, PR China; State Key Laboratory of Marine Resource Utilization in South China Sea, Hainan University, Haikou, Hainan 570228, PR China

**Keywords:** siderophore receptors, *V*ibrio alginolyticus, *B*acillus velezensisbacillibactin, siderophore piracy

## Abstract

The opportunistic pathogen *Vibrio alginolyticus* dominates iron-depleted marine ecosystems, likely driven by its diverse repertoire of siderophore receptors that enable iron piracy from exogenous sources. While the ability to utilize xenosiderophores via piracy can be advantageous under iron limitation, the identities of exogenous siderophore producers interacting with *V. alginolyticus* remain poorly characterized. Here, we show that 17.0% of siderophore-producing isolates from *V. alginolyticus*-dominated mariculture systems significantly enhance the growth of *V. alginolyticus* HN08155 under iron limitation, including six *Bacillus* strains established as probiotics in aquaculture. Notably, *Bacillus velezensis* WD26-16 exhibits the strongest growth-promoting effect via catechol-type siderophore bacillibactin production. Genomic analyses demonstrate that 86.1% of marine *Bacillus* spp. in the Genome Taxonomy Database harbor conserved bacillibactin biosynthetic gene clusters, with near-complete conservation across all *B. velezensis* strains, suggesting ubiquitous siderophore-mediated interaction with *V. alginolyticus*. Exogenous bacillibactin induces distinct metabolic modulation in *V. alginolyticus*, activating pathways critical for amino acid metabolism, protein biosynthesis, and energy production to sustain proliferative demands. This metabolic adaptation is mediated by coordinated upregulation of multiple siderophore receptors (IutA, IrgA, VctA) that allows functional plasticity in xenosiderophore piracy. Co-culture experiments reveal that *V. alginolyticus* exploits bacillibactin to outcompete *B. velezensis* and achieves a 3.4-fold growth advantage compared to the monoculture. Our results uncover an ecological paradox: probiotic *B. velezensis* inadvertently enhances pathogenic *V. alginolyticus* proliferation through siderophore piracy. This iron-centric competition mechanism likely drives vibriosis outbreaks in aquaculture systems, necessitating urgent reassessment of probiotic selection criteria to avoid unintended pathogen amplification.

## Introduction

Iron is an essential micronutrient in marine ecosystems, yet its bioavailability is severely constrained by oxidative conditions that limit dissolved iron concentrations to levels below the growth requirements of most bacteria [1, 2]. This scarcity drives fierce interspecies competition among marine bacteria, prompting the evolution of specialized iron-scavenging strategies. A key adaptation involves siderophores—high-affinity Fe(III)-chelators that mobilize and assimilate bioavailable iron from the environment [3]. This siderophore-mediated iron acquisition system represents a critical survival strategy for marine bacteria to improve competitive fitness under iron limitation [4, 5].

The genus *Vibrio* represents a paradigm of ecological versatility among marine bacteria, leveraging genomic plasticity and opportunistic heterotrophy to thrive across fluctuating environmental conditions [6]. Their ecological success is underpinned by a broad genomic and metabolic repertoire, enabling them to achieve dominance in diverse niches ranging from oligotrophic marine environments to pathogenic host associations [7]. A notable feature of iron acquisition in *Vibrio* is the uneven distribution of siderophore production capability within populations. Even among very closely related strains, only ~40% on average are producers. Nonproducers evolved by a selective loss of only the biosynthesis genes but retention of the associated receptor, allowing a cheating behavior for iron acquisition [8]. Notably, the number of siderophore receptors encoded in a *Vibrio* genome typically exceeds the number of biosynthetic clusters [9], suggesting that siderophore cheating, which involves the exploitation of heterologous siderophores produced by co-occurring microbes, is common in *Vibrio* [5, 10]. This adaptive trait is exemplified by *V. cholerae*, which employs receptors IrgA and VctA to scavenge enterobactin from *Escherichia coli*, ViuA and VctA to pirate fluvibactin from *V. fluvialis*, and FhuA to assimilate ferrichrome [11, 12]. Such siderophore receptor-mediated cheating may confer ecological advantages, allowing bacteria to bypass metabolic costs of siderophore production while capitalizing on microbial public goods.

Emerging studies of rhizosphere and gut microbiomes reveal that pathogens can hijack compatible siderophores synthesized by resident microbial members, using them to outcompete rivals. This cross-species siderophore sharing inadvertently fuels pathogen expansion [13, 14]. Such siderophore-mediated interactions significantly contribute and reliably predict pathogen growth and disease outcomes in many cases, establishing a key framework for understanding pathogenesis in terrestrial ecosystems [15]. This paradigm also offers novel insights into the ecological adaptability of marine pathogens.

The evolutionary arms race in siderophore exploitation is mechanistically linked to the genomic diversity of receptor repertoires [16]. Pathogenic *Vibrio* species typically possess multiple siderophore receptors, with *V. alginolyticus* strains encoding up to seven to eight distinct receptors in a single genome—a relatively high number within the *Vibrio* genus [9]. As a dominant pathogen in mariculture and coastal ecosystems, *V. alginolyticus* has been shown to proliferate explosively in response to various environmental factors (e.g. temperature, salinity, inorganic and organic nutrients) [17, 18]. While studies have delineated intrinsic adaptive mechanisms such as quorum sensing regulatory circuits, RpoN-dependent stress responses, and versatile metabolic plasticity (e.g. Tricarboxylic Acid Cycle (TCA) pathway–mediated adhesion) [19, 20], critical knowledge gaps persist regarding its ecological dominance in marine niches. Notably, *V. alginolyticus* rapidly proliferates in response to Saharan dust–derived iron [2], implicating efficient iron acquisition as a key survival strategy. However, despite genomic evidence of enriched siderophore receptors in *V. alginolyticus*, it remains unclear whether these receptors facilitate siderophore piracy from cohabiting microbes as a competitive adaptation strategy.

The following critical scientific questions remain unresolved: (i) From the perspectives of microbial taxonomy and siderophore types, what kinds of siderophores secreted by which microorganisms can be pirated by *V. alginolyticus*? (ii) Which specific siderophore receptor(s) mediate this xenosiderophore piracy process in *V. alginolyticus*? (iii) What survival or adaptive advantages does *V. alginolyticus* gain through siderophore piracy in iron-limited environments? To address these knowledge gaps, we isolated a diverse collection of siderophore-producing bacteria from marine aquaculture systems where *V. alginolyticus* is prevalent. We conducted supernatant feeding assays under iron-limited culture conditions to systematically investigate the effects of these siderophore producers on the pathogenic *V. alginolyticus* strain HN08155, which we previously isolated from mariculture ecosystems. Notably, genomic analysis revealed that this pathogen encodes at least seven distinct siderophore receptors, highlighting its diverse iron piracy mechanisms and making it an ideal model for studying siderophore-mediated microbial interactions in marine ecosystems.

Here, we report a previously unrecognized ecological interaction wherein the probiotic *Bacillus* promotes the proliferation of the opportunistic pathogen *V. alginolyticus* through siderophore piracy. Molecular characterization reveals that *V. alginolyticus* HN08155 employs multiple siderophore receptors (IutA, IrgA, VctA) to hijack bacillibactin, a catecholate siderophore produced by *B. velezensis*. Intriguingly, phylogenomic evidence demonstrates the ubiquitous distribution and evolutionary conservation of bacillibactin biosynthesis gene clusters among marine *Bacillus* strains, suggesting that this piracy mechanism may represent a prevalent ecological strategy for *V. alginolyticus* in iron-limited marine environments. This study broadens current perspectives on microbial interactions between two ecologically dominant bacterial genera in marine aquaculture environments.

## Materials and methods

### Bacterial growth conditions

*Vibrio alginolyticus strain* HN08155 and its derivative strains were routinely cultured in 2216E medium (5 g/l peptone, 1 g/l yeast extract, and 30 g/l NaCl) at 30°C. To establish culture conditions with different degrees of iron limitation, the well-characterized iron chelator 2,2′-bipyridine (Dip) was supplemented into 2216E medium at varying concentrations. For the supernatant feeding assay, iron-limited M9 medium was prepared by dissolving 1 g NH_4_Cl, 3 g KH_2_PO_4_, and 20 g NaCl in MilliQ water, followed by pH adjustment to 6.8 using 10 M NaOH. The solution was brought to 960 ml, sterilized by autoclaving, and supplemented after cooling with 30 ml of sterile 10% (w/v) casamino acids and 10 ml of sterile 20% (w/v) glucose. For genetic manipulation, E*.* coli strains DH5α and β2163 were grown in Luria-Bertani (LB) medium (10 g/l tryptone, 5 g/l yeast extract, and 10 g/l NaCl) at 37°C, with strain β2163 additionally supplemented with 0.3 mM diaminopimelic acid (DAP) during cultivation. Solid medium was prepared by adding 1.5% (w/v) agar. Antibiotics were added as required at the following final concentrations: 100 μg/ml ampicillin (Amp) and 25 or 50 μg/ml chloramphenicol (Cm). Bacterial strains and plasmids used in this study are listed Supplementary Table S1.

### Growth dynamics of *V. alginolyticus* HN08155 under varying iron availability

Cultures of *V. alginolyticus* HN08155 were established in 2216E liquid medium supplemented with incremental concentrations of the iron-chelating agent Dip (50, 100, 150, and 200 μM). To assess iron-dependent growth recovery, a parallel iron-restored condition was created by supplementing 200 μM FeCl_3_ into 2216E medium containing 200 μM Dip. All experimental groups were incubated at 30°C with continuous agitation (180 rpm) over a 24-h period. The bacterial growth dynamics were monitored using an automated microplate reader, with optical density at 600 nm (OD_600_) measured at regular intervals to generate comparative growth profiles through real-time data acquisition.

### Isolation and identification of siderophore-producing strains

Samples of water and shrimp were collected from *Litopenaeus vannamei* aquaculture systems located in Hainan, Guangdong, and Guangxi provinces during March to May 2023. Following standard pretreatment procedures, diluted samples were spread onto 2216E agar plates and incubated at 30°C for 48 h to obtain pure bacterial isolates. The purified strains were subsequently assayed for siderophore production via the Chrome azurol S (CAS) agar plate method following established protocols [8]. Briefly, each strain was inoculated into 2216E liquid medium and cultured under shaking conditions (30°C, 24 h). Bacterial cells were harvested by centrifugation (4000 rpm, 10 min), then resuspended in iron-limited 2216E medium (containing 200 μM Dip) for an additional 24-h incubation. After centrifugation, the pellet was resuspended in 200 μl of iron-limited medium, and 50 μl aliquots were inoculated into wells on CAS agar plates. Following 24-h incubation at 30°C, siderophore production was confirmed by the formation of yellow or orange halos around the wells, indicative of iron-chelating activity. Siderophore-producing strains were identified via PCR amplification of 16S rDNA using universal primers 27F (5′-TACGGCTACCTTGTTACGACTT-3′) and 1492R (5′-AGAGTTTGATCCTGGCTCAG-3′). Sequencing results were analyzed using the National Center for Biotechnology Information (NCBI) and EzBioCloud databases to determine homologous sequence similarities.

### Supernatant feeding assay

The siderophore-producing bacterial strains were grown in iron-limited 2216E liquid medium added with 200 μM Dip under shaking conditions (180 rpm) at 30°C for 48 h. After cultivation, the bacterial suspension was centrifuged at 10 000 rpm for 10 min, and the resulting supernatant was filter-sterilized through a 0.22 μm membrane to obtain cell-free culture supernatant. For the supernatant feeding assay, 20 μl of the sterile supernatant was mixed with 180 μl of iron-limited M9 medium in a 96-well plate. Subsequently, 2 μl of *V. alginolyticus* HN08155 culture was inoculated into each well. The plate was incubated with continuous shaking (180 rpm) at 30°C. Sterilized distilled water served as a blank control, while the cell-free supernatant from the iron-limited cultured strain HN08155 was used as a negative control. Bacterial growth dynamics were monitored over 24 h using a microplate reader, with biomass quantified by measuring OD_600_. The experimental workflow is illustrated in Supplementary Fig. S1.

### Crude siderophore extraction and bioactivity assay

The cell-free supernatant of the siderophore-producing strain WD26-16 was mixed with ethyl acetate in a 5:1 (v/v) ratio in a separatory funnel. The mixture was vortexed vigorously for 5 min and allowed to phase-separate for 5 min. The aqueous phase was retained, and the extraction process was repeated three times under identical conditions to maximize yield. The pooled organic phase was concentrated to dryness using a rotary evaporator at 40°C, and the residue was re-suspended in a minimal volume of ultrapure water before lyophilization to obtain the crude siderophore extract. A working solution (1 mg/ml) was prepared by dissolving the lyophilized siderophore extract in sterile deionized water, followed by filter sterilization through 0.22 μm membranes for subsequent experimental use.

For the solid version of the CAS assay, 50 μl of the aqueous crude extract was loaded into wells on CAS agar plates. Characteristic yellow-to-orange halo zones surrounding the wells within 1–2 h of incubation indicated siderophore production. For the liquid version of the CAS assay, 99 μl of the crude extract was combined with 99 μl CAS dye reagent and 2 μl shuttle solution, followed by a 15-min incubation in darkness. A chromatic transition from blue to yellow/orange confirmed siderophore activity, with reagent preparation following established protocols [8]. To validate the growth enhancement effect of the crude extract on *V. alginolyticus* HN08155, 20 μl of the sterile crude siderophore extract was added to 180 μl of iron-limited M9 medium in a 96-well microplate. The mixture was inoculated with 2 μl of *V. alginolyticus* HN08155 preculture and incubated at 30°C with agitation (180 rpm) for 24 h. Bacterial growth kinetics were monitored at 600 nm using a microplate reader.

### Determination of siderophore type and structural characterization

The siderophore type secreted by strain WD26-16 was determined using the Arnow test and ferric perchlorate assay [21]. For the Arnow test, reaction solutions were prepared by dissolving 10 g each of NaNO_2_ and Na_2_MoO_4_·2H_2_O in 50 ml of deionized water. A 3 ml aliquot of crude siderophore extract was mixed with 0.1 ml of 5 M HCl and 0.5 ml of the reaction solution. Upon yellow coloration, 0.1 ml of 10 M NaOH was added and vortexed thoroughly. A persistent red coloration indicated the presence of catechol-type siderophores. For the ferric perchlorate assay, 0.5 ml of crude siderophore extract was combined with 2.5 ml of ferric perchlorate solution, with immediate red-orange coloration indicating hydroxamate-type siderophores.

Structural characterization was performed using an Agilent 1290 Infinity II-6460 Liquid Chromatography–Tandem Mass Spectrometry (LC–MS/MS) system (Santa Clara, CA, USA). Chromatographic separation was performed on an Agilent SB-C18 column (2.1 × 100 mm, 1.8 μm) maintained at 30°C. The mobile phase comprised 0.1% formic acid in water (phase A) and acetonitrile (phase B), with the following gradient: 0–0.5 min (5% B), 0.5–15 min (5%–95% B), 15–20 min (95% B), and 20.01–24 min (5% B) for equilibration. The flow rate was 0.2 ml/min, with a 5 μl injection volume and autosampler temperature of 20°C. Mass spectrometry parameters included an Electrospray Ionization (ESI) ion source in negative ionization mode (m/z range: 100–1000), source temperature of 350°C, nebulizer gas flow rate of 10 L/min, nebulizer pressure of 45 psi, capillary voltage of 3500 V, and fragmentor voltage of 80 V.

### Identification of the siderophore biosynthetic gene cluster

Genomic DNA was extracted from strain WD26-16 using a bacterial genomic DNA extraction kit (Omega Bio-Tek, Guangzhou, China). The *dhbA*, *dhbB*, and *dhbC* genes were amplified via PCR with specific primers (Supplementary Table S2). PCR amplicons were analyzed by agarose gel electrophoresis to verify target band sizes. Fragments of expected lengths were purified and subjected to Sanger sequencing (BioSune, Shanghai, China) using the same primers as for amplification. The obtained sequences were aligned using the NCBI Blastx database to preliminarily identify putative siderophore synthase genes. Whole-genome sequencing was performed using PacBio SMRT (Single-Molecule Real-Time) technology at Biomarker Technologies (Beijing, China). Raw sequencing data were filtered to remove low-quality reads and assembled using Hifiasm (v0.16.1). The assembly was optimized and error-corrected with Pilon (v1.22) to generate a high-quality genome sequence. Functional annotation of the assembled genome was performed using the Non-Redundant Protein Sequence Database (NR), Gene Ontology (GO), Kyoto Encyclopedia of Genes and Genomes (KEGG), Clusters of Orthologous Groups (COG), Swiss-Prot, and Pfam databases. Secondary metabolite biosynthetic gene clusters (BGCs) were systematically analyzed via the antiSMASH online platform (https://antismash.secondarymetabolites.org/), specifically targeting siderophore BGCs.

### Phylogenetic analyses of bacillibactin biosynthetic gene clusters from marine *Bacillus* genomes

Marine-derived *Bacillus* genomes were systematically curated from the Genome Taxonomy Database (GTDB Release 09-RS220; https://gtdb.ecogenomic.org/) through a targeted keyword search incorporating marine habitat descriptors (“sea,” “marine,” “ocean,” “estuary,” “tidal,” “reef,” “mangrove,” and “coast”). Corresponding amino acid FASTA files for these genomes were retrieved from the NCBI RefSeq database and compiled into a curated local protein database using BioEdit software (v7.0.5.2) [22]. Reference amino acid sequences of bacillibactin BGC from strain WD26-16 served as queries for local BLASTp analysis against the constructed marine *Bacillus* protein database. High-stringency alignment parameters were applied (E-value ≤ 1.0E−40) to ensure precise identification of homologous BGCs, leveraging stringent sequence similarity thresholds to minimize false positives. For phylogenetic reconstruction, DhbF protein sequences were aligned using the MUSCLE algorithm in MEGA X (v10.0.5) with default parameters [23]. A Maximum Likelihood tree was generated using the Jones-Taylor-Thornton (JTT) substitution matrix, incorporating gamma-distributed rate variation among sites and a proportion of invariant positions. Topological robustness was assessed through 1000 bootstrap replicates. The resulting phylogenetic tree was visualized and annotated using the iTOL platform (https://itol.embl.de/).

### Mutant construction

The siderophore receptor gene knockout was performed using a two-step allelic exchange strategy as previously described [24]. For constructing deletion mutants of target genes in *V. alginolyticus*, flanking regions (~600 bp each) upstream and downstream of the desired gene were PCR-amplified from genomic DNA using specific primers (Supplementary Table S2). These fragments were fused through overlap extension PCR and subsequently cloned into the suicide vector pDM4 via restriction enzyme digestion and T4 DNA ligase-mediated ligation. The recombinant plasmid was introduced into *V. alginolyticus* via conjugation with *E. coli* β2163 (a DAP-auxotrophic donor strain harboring the *pir* gene). Transconjugants were selected on Luria-Bertani Salt (LBS) agar plates (LB supplemented with 30 g/l NaCl) supplemented with ampicillin (100 μg/ml) and chloramphenicol (25 μg/ml). Positive clones undergoing homologous recombination were counter-selected using 10% sucrose-containing medium to eliminate the *sacB*-containing plasmid backbone. Successful gene deletion was verified through PCR amplification and sequencing analysis.

### Effect of bacillibactin on the growth of HN08155 on iron-limited agar plates

*Vibrio alginolyticus* HN08155 was cultured in 2216E liquid medium at 30°C with continuous shaking (180 rpm) until reaching mid-exponential growth phase (OD_600_ = 0.5–0.6). Bacterial suspensions were serially diluted (10-fold gradients from 10^−1^ to 10^−4^), and 100 μl aliquots from each dilution were spread onto iron-limited 2216E agar plates supplemented with 200 μM Dip. For experimental treatments, three sterile filter paper discs impregnated with 20 μl of crude bacillibactin extract were equidistantly positioned on the agar surface inoculated with strain HN08155. Control plates received disks loaded with an equivalent volume of sterile deionized water. Triplicate plates were prepared for both treatment and control groups at each dilution gradient. All plates were incubated at 30°C for 24 h, and bacterial growth was qualitatively assessed by macroscopic examination of colony formation and density.

Following incubation, bacterial biomass was aseptically harvested from the agar surfaces using sterile cell scrapers and immediately transferred into pre-chilled, nuclease-free 1.5 ml microcentrifuge tubes. Samples were flash-frozen in liquid nitrogen and stored at −80°C prior to downstream RNA extraction and transcriptional profiling.

### RNA extraction and transcriptome sequencing

Total RNA extraction was performed using the Eastep® Super Total RNA Extraction Kit (Promega, Shanghai, China), followed by quality assessment through NanoPhotometer® (Implen, Germany). Qualified RNA samples were processed for RNA sequencing on an Illumina NovaSeq 6000 platform (San Diego, CA, USA) at Majorbio Bio-Pharm Technology (Shanghai, China). Raw sequencing reads underwent quality control using Fastp (v0.23.2) to remove adapters and low-quality reads. Clean reads were aligned to the *V. alginolyticus* HN08155 reference genome using HISAT2 (v2.2.1), followed by transcript assembly via StringTie (v2.2.1). Differential expression analysis was performed using DESeq2 (v1.38.3) with thresholds of |log_2_(fold change)| ≥ 1.0 and adjusted *P*-value < .05. Significant differentially expressed genes (DEGs) were functionally annotated through BLASTp searches against the NCBI NR database and subjected to enrichment analysis using KEGG and GO databases via the Majorbio Cloud Platform (https://cloud.majorbio.com/).

### Quantitative real-time PCR analysis

The expression profiles of siderophore receptor genes under different experimental conditions (with or without bacillibactin supplementation) or across bacterial strains (wild-type and mutant variants of V. alginolyticus) were analyzed by quantitative real-time PCR (qPCR). Total RNA samples meeting quality control criteria were reverse-transcribed into first-strand complementary DNA (cDNA) using the HiScript® II Q RT SuperMix for qPCR (+gDNA wiper) (Vazyme, Nanjing, China). Subsequent qPCR amplifications were performed in triplicate on a LightCycler® 480 real-time PCR system (Roche, Basel, Switzerland) using ChamQ Universal SYBR qPCR Master Mix (Vazyme). Each 20 μl reaction contained 10 μl of 2× SYBR mix, 0.4 μl each of forward and reverse primers (final concentration 200 nM), and 2 μl cDNA template. Relative gene expression levels were normalized against the housekeeping gene *gyrB* and calculated using the 2^−ΔΔCt^ method [25], with data derived from three independent biological replicates. Primers sequences are detailed in Supplementary Table S2.

### Coculture experiment

*Vibrio alginolyticus* HN08155 and *B. velezensis* WD26-16 were individually cultured in 2216E liquid medium at 30°C with shaking (180 rpm) until reaching an OD_600_ of 0.6. Colony-forming units (CFUs) were quantified via serial dilution and plate counting, revealing that OD_600_ = 0.6 corresponded to 2 × 10^8^ CFU/ml for HN08155 and 2.5 × 10^7^ CFU/ml for WD26-16. For the coculture group (VB group), 250 μl of HN08155 culture (5 × 10^7^ CFU) and 2 ml of WD26-16 culture (5 × 10^7^ CFU) were mixed and cocultured in iron-limited 2216E medium (containing 200 μM Dip). A monoculture control (V group) containing 250 μl of HN08155 culture was prepared under identical iron-limited conditions. Both experimental and control groups were conducted in biological triplicate and incubated at 30°C with shaking (180 rpm) for 24 h.

After incubation, bacterial cultures from the VB and V groups were subjected to 10-fold serial dilutions (up to 10^−5^). Aliquots (100 μl) of each dilution were spread onto both nonselective 2216E agar and *Vibrio*-selective Thiosulfate Citrate Bile Salts Sucrose (TCBS) agar, with triplicate plates per dilution. After overnight incubation at 30°C, *V. alginolyticus* colonies were quantified through distinctive yellow colony enumeration on TCBS agar plates, enabling comparative analysis between VB and V groups. To quantify the HN08155/WD26-16 ratio in the VB group, 100 randomly selected colonies from 2216E agar plates were subjected to PCR amplification using HN08155-specific primers (Supplementary Table S2). Correctly sized amplicons on agarose gel electrophoresis confirmed HN08155, while no bands indicated WD26-16. The fitness of strain HN08155 relative to strain WD26-16 was calculated using the formula *w* = (*A*_t_ × *B*_0_)/(*A*_0_ × *B*_t_), where *A*_0_ and *B*_0_ represent initial population frequencies of strain HN08155 and WD26-16, respectively, and *A*_t_ and *B*_t_ reflect final frequencies [26].

### Statistical analysis

The statistical analyses were conducted using the SPSS 18.0 statistical software. All experimental assays were performed in triplicate, and the results were expressed as mean ± standard deviation. Statistical significance was assessed using paired two-tailed Student’s *t*-tests.

## Results

### Phylogenetically diverse siderophore-producers rescue iron-restricted growth of *V. alginolyticus* HN08155

The growth kinetics of *V. alginolyticus* HN08155 exhibited dose-dependent sensitivity to iron limitation imposed by 2,2′2 208 155 exh (Dip) in 2216E medium. While 50 μM and 100 μM Dip caused moderate biomass reductions (0.16 ± 0.03 and 0.29 ± 0.05, respectively) at 24 h, 150 μM Dip triggered severe growth suppression (biomass decrease: 0.62 ± 0.07). At 200 μM Dip, the growth of strain HN08155 was drastically inhibited, resulting in only minimal yet detectable biomass accumulation. This nearly complete growth suppression was fully reversed upon supplementation with 200 μM FeCl₃ (Supplementary Fig. S2), thereby confirming iron limitation as the primary constraint on growth.

**Figure 1:**
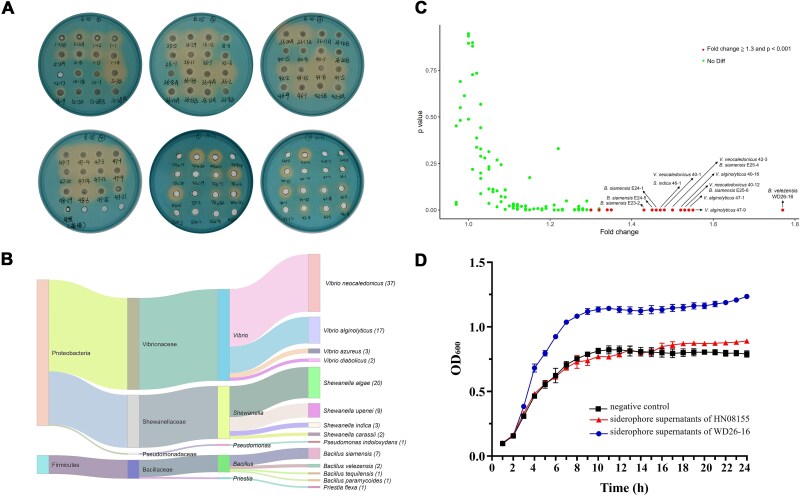
Isolation of siderophore-producing bacteria and their effects on the growth of *V. alginolyticus* HN08155 under iron-limited conditions. (A) Siderophore production by representative strains using the CAS plate assay. (B) Sankey diagram of phylogenetic distribution of siderophore-producing bacterial isolates. (C) Volcano plot showing the effects of siderophore-enriched supernatants on the growth of *V. alginolyticus* under iron-limited conditions. Red dots: strains with significant growth enhancement (fold change ≧ 1.3, *P* < .001); green dots: nonsignificant effects. (D) Growth kinetics of *V. alginolyticus* in iron-limited medium supplemented with siderophore supernatant from strain WD26-16.

To identify bacterial taxa capable of producing growth-promoting siderophores that enhance the growth of *V. alginolyticus* HN08155 under iron-limited conditions, we isolated 1011 bacterial strains from mariculture systems in Hainan, Guangdong, and Guangxi provinces. Through CAS plate screening (Fig. 1A), 106 strains (10.5% of total isolates) exhibited siderophore production activity (Supplementary Table S3). 16S rDNA-based phylogenetic analysis classified these siderophore producers into two phyla (Proteobacteria and Firmicutes), four families (Vibrionaceae, Shewanellaceae, Bacillaceae, and Pseudomonadaceae), and five genera (*Vibrio*, *Shewanella*, *Bacillus*, *Pseudomonas*, and *Priestia*). Among these, three genera emerged as predominant siderophore producers, with *Vibrio* being the most prevalent (59 strains, 55.7% of total siderophore-producing strains), followed by *Shewanella* (34 strains, 32.1%) and *Bacillus* (11 strains, 10.4%) (Fig. 1B).

We then conducted a supernatant feeding assay to assess the role of bacterial siderophores in the growth of *V. alginolyticus* HN08155 under iron-limited conditions (Supplementary Fig. S1). Siderophore-enriched supernatants from 106 producer strains were introduced into HN08155 cultures, revealing that 18 strains (17.0%) significantly enhanced its growth (fold change ≥1.3 with *P* < .001; Supplementary Table S3, Supplementary Fig. S3). Notably, eight strains exhibited a pronounced ability to rescue the growth deficiency of HN08155 via their siderophore-enriched supernatants. These growth-promoting siderophore producers comprised three *Bacillus* strains (*B. velezensis* WD26-16, *B. siamensis* E25-4 and E25-6) and five *Vibrio* strains (*V. alginolyticus* 40-16, 47-1 and 47-9, and *V. neocaledonicus* 40-12 and 42-3). These strains mediated a >1.5-fold increase in biomass compared to siderophore-free control after 24 h of cultivation (Fig. 1C), supporting the hypothesis of cross-strain siderophore piracy, though contributions from other metabolites in the supernatants cannot be entirely excluded.

The genome of *V. alginolyticus* HN08155 harbors the gene clusters *pvsABCDE* and *psuA*-*pvuABCDE* (Supplementary Fig. S4A), which are predicted to be responsible for the biosynthesis and transport of the siderophore vibrioferrin based on protein alignment [27]. Repeated CAS plate assays consistently detected siderophore production in this strain, though at low levels (Supplementary Fig. S4B). To assess the growth-promoting capacity of this endogenous siderophore, we collected the cell-free supernatant of strain HN08155 cultured under iron-limited conditions and introduced it into fresh iron-limited medium to re-cultivate HN08155. However, this supplementation did not result in a significant difference in the growth of HN08155 compared to the negative control (Fig. 1D). This indicates that the siderophore produced by strain HN08155 exhibits insufficient iron-scavenging activity to confer a competitive advantage under the specific laboratory conditions, which is supported by the constrained growth of strain HN08155 in 2216E medium added with 200 μM Dip (Supplementary Fig. S2).

Remarkably, supplementation with *B. velezensis* WD26-16-derived supernatant elicited the most robust growth stimulation of *V. alginolyticus* HN08155 among all 106 siderophore-producing strains tested (Fig. 1D, Supplementary Fig. S3). In view of the ecological dominance of *Vibrio* and the ecological importance of *Bacillus* in many marine environments, and given the scarcity of studies on their siderophore-mediated cross-genus interactions, we focused on deciphering the molecular basis of this iron piracy phenomenon. Using strain WD26-16 as a model siderophore-producer, we systematically characterized the chemical identity of its bioactive compound(s) and their interaction mechanisms with *V. alginolyticus* HN08155.

### Catecholic siderophore bacillibactin from *B. velezensis* WD26-16 fuels *V. alginolyticus* HN08155 proliferation

**Figure 2:**
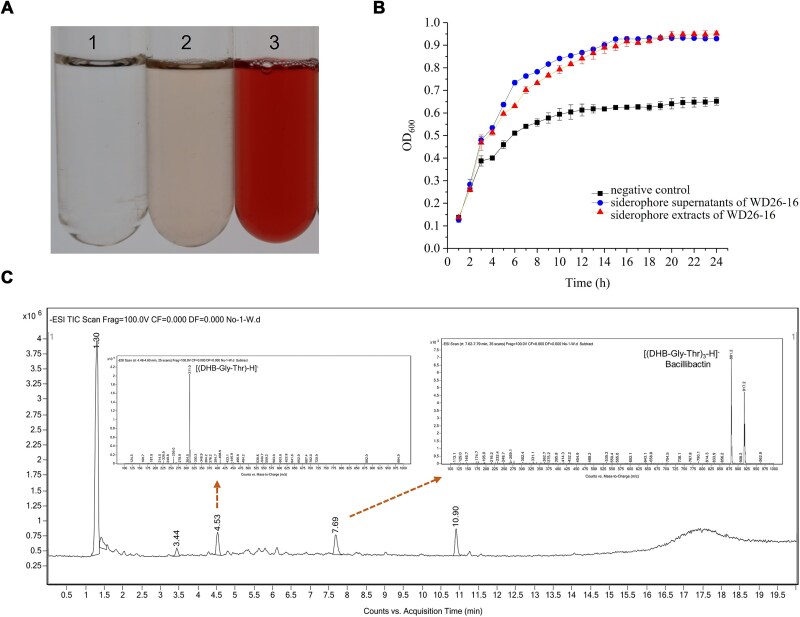
Identification and characterization of siderophores secreted by *B. velezensis* strain WD26-16. (A) Arnow assay detecting catechol-type siderophore in crude extracts: tube 1 (sterile water), tube 2 (iron-limited medium), tube 3 (crude extracts from WD26-16). (B) Effect of crude siderophores from strain WD26-16 on the growth of *V. alginolyticus* HN08155 under iron-limiting conditions. (C) HPLC chromatogram and mass spectrum of the siderophore bacillibactin.

The cell-free supernatant of strain WD26-16, cultured under iron-limited conditions, was extracted for systematic siderophore profiling. The presence of siderophores in the crude extracts was confirmed by CAS assays, which exhibited characteristic yellow halos in plate assays (well 3, Supplementary Fig. S5A) and distinct orange chromogenic shifts in solution assays (well 3, Supplementary Fig. S5B). Chemical analysis further identified the siderophore as catechol-type, as evidenced by Arnow’s test, which produced an intense diagnostic red coloration (tube 3, Fig. 2A), while the ferric perchlorate assay remained colorless (tube 3, Supplementary Fig. S5C). Functional validation was achieved through siderophore supplementation assays, demonstrating significant growth promotion of *V. alginolyticus* HN08155 in iron-depleted media. Both crude extracts and siderophore-enriched supernatant from WD26-16 showed similar growth enhancement effects (Fig. 2B), demonstrating the critical role of this catecholic siderophore in supporting the growth of *V. alginolyticus* under iron limitation.

Catecholic siderophores are typically synthesized using 2,3-dihydroxybenzoic acid (DHB) as a precursor [28]. To validate the genetic potential of strain WD26-16 for catecholic siderophore production, we amplified the genes *dhbA*, *dhbB*, and *dhbC*, which encode conserved enzymes involved in DHB biosynthesis. This step was conducted prior to whole-genome sequencing to rapidly confirm the presence of core biosynthetic machinery and to guide subsequent chemical analyses. PCR analysis using established primers confirmed that strain WD26-16 possesses three essential DHB biosynthesis genes (Supplementary Fig. S5D). BLASTx annotation revealed that these three fragments encode 2,3-dihydro-2,3-dihydroxybenzoate dehydrogenase (DhbA), isochorismatase (DhbB), and isochorismate synthase (DhbC), respectively. High-Performance Liquid Chromatography (HPLC)-ESI-MS characterization revealed the siderophore profile of WD26-16. While five chromatographic peaks were detected, MS signatures at 4.53 min (m/z 311.0) and 7.69 min (m/z 881.2) specifically indicated catechol-type siderophores (Fig. 2C). The mass peak at m/z 881.2 corresponds to the cyclic catecholic siderophore bacillibactin, which consists of three DHB-Gly-Thr units, i.e. [(DHB-Gly-Thr)_3_-H]^−^. Meanwhile, the mass peak at m/z 311.0 matches its hydrolytic monomer [(DHB-Gly-Thr)-H]^−^. These findings demonstrate that *B. velezensis* WD26-16 significantly promotes the growth of *V. alginolyticus* HN08155 under iron-limited culture conditions through secretion of the catechol-type siderophore bacillibactin.

### Bacillibactin biosynthetic gene clusters are ubiquitous in marine *Bacillus* species

Through integrated whole-genome sequencing and functional annotation analyses, we identified the complete BGC governing bacillibactin production in strain WD26-16 (Supplementary Fig. S6A, Supplementary Table S4). By coupling KEGG pathway mapping with established biochemical frameworks of bacillibactin synthesis, we reconstructed the bacillibactin biosynthetic pathway in WD26-16. The pathway initiates with chorismate, which is converted into isochorismate by the isochorismate synthase (DhbC). Isochorismatase (DhbB) then hydrolyzes this intermediate to generate 2,3-dihydro-2,3-dihydroxybenzoate, which undergoes dehydrogenation via DhbA to yield the siderophore precursor 2,3-dihydroxybenzoate (DHB). Finally, DHB is conjugated with glycine (Gly) and threonine (Thr) through peptide bond formation, a process catalyzed by nonribosomal peptide synthetase (DhbE, DhbB, and DhbF), leading to the synthesis of bacillibactin (Supplementary Fig. S6B).

**Figure 3:**
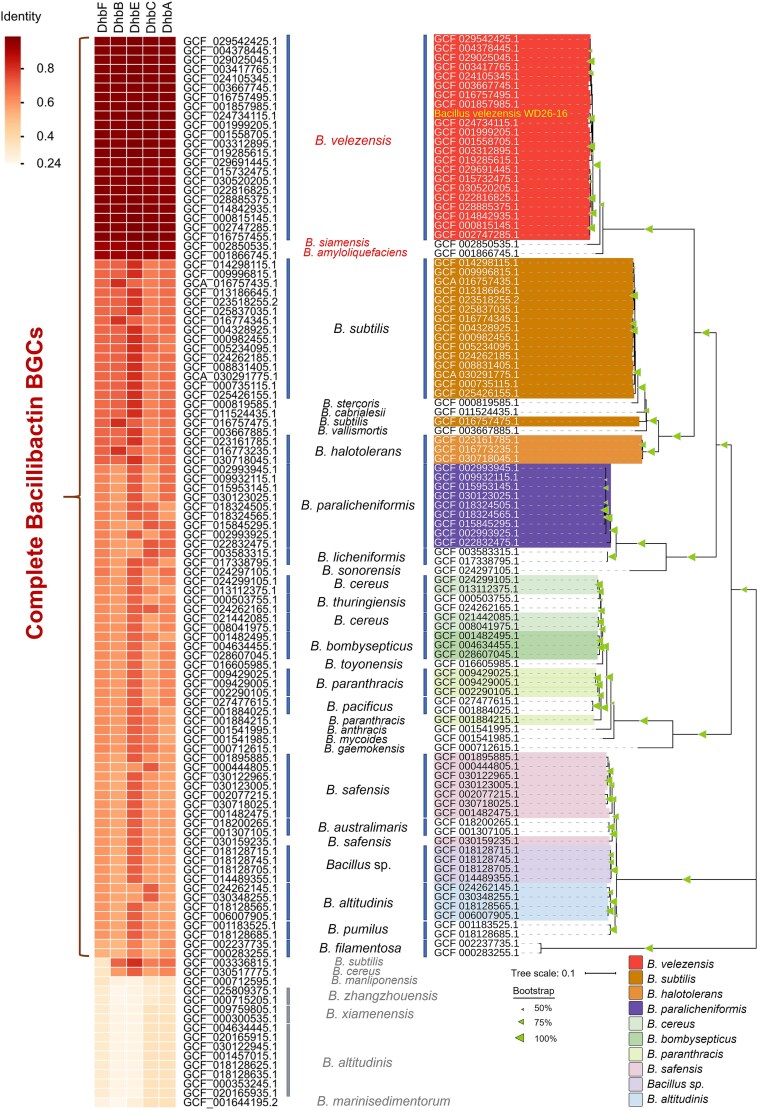
Phylogenomic distribution of bacillibactin BGCs across 115 marine-derived *Bacillus* genomes from the GTDB database. Left panel: Heatmap depicting gene-wise amino acid similarity (%) calculated by BLASTp. Middle panel: Taxonomic assignments of strains. Right panel: Maximum-likelihood phylogenetic tree of DhbF synthetase homologs, with bootstrap values exceeding 50% (indicated at nodes). Genome accession numbers (NCBI source) are provided for each strain.

While bacillibactin biosynthesis is well documented in terrestrial *Bacillus* species [29], its ecological prevalence and evolutionary conservation in marine *Bacillus* populations remain systematically uncharacterized. To investigate whether bacillibactin production is a common trait within marine *Bacillus* species, we systematically analyzed bacillibactin BGCs across all marine-derived *Bacillus* genomes in the GTDB. Strikingly, 86.1% (99/115) of marine *Bacillus* genomes harbored complete bacillibactin BGCs, with core genes sharing >57% average amino acid identity (AAI) to WD26-16 homologs (Fig. 3, Supplementary Table S5). Phylogenetic analysis of DhbF, the key enzyme responsible for the terminal step in bacillibactin biosynthesis, demonstrated distinct species-specific clustering patterns among marine *Bacillus* strains. Homologous DhbF sequences from conspecific strains predominantly grouped together (Fig. 3), indicating strong evolutionary conservation of this siderophore synthesis machinery at the taxonomic species level. This observation aligns with recent findings showing a positive association between BGC distance and phylogenetic distance within the genus *Bacillus* [30].

Notably, all *B. velezensis* isolates (22/22) retained complete bacillibactin BGCs exhibiting exceptional conservation, exhibiting over 97% average AAI to strain WD26-16. The DhbF sequences from these *B. velezensis* strains displayed near-perfect phylogenetic congruence, indicating a highly pervasive conservation of bacillibactin. Furthermore, significant phylogenetic proximity and high sequence identity (97%) were observed between the DhbF sequences of *B. velezensis* and those of representative strains of *B. siamensis* and *B. amyloliquefaciens* (Fig. 3, Supplementary Table S5). This suggests potential functional conservation of bacillibactin-mediated iron acquisition systems across these *Bacillus* species. Collectively, these findings demonstrate that bacillibactin BGCs are ubiquitously conserved in marine *Bacillus* species, with *B. velezensis* strains showing nearly identical genetic architectures to WD26-16. This conservation implies that the facilitation of *V. alginolyticus* growth under iron-limited conditions by bacillibactin may represent a prevalent ecological adaptation in marine ecosystems, particularly in interactions between *B. velezensis* and *V. alginolyticus*.

### Functionally overlapping siderophore receptor arsenal empowers *V. alginolyticus* HN08155 to pirate bacillibactin

**Figure 4:**
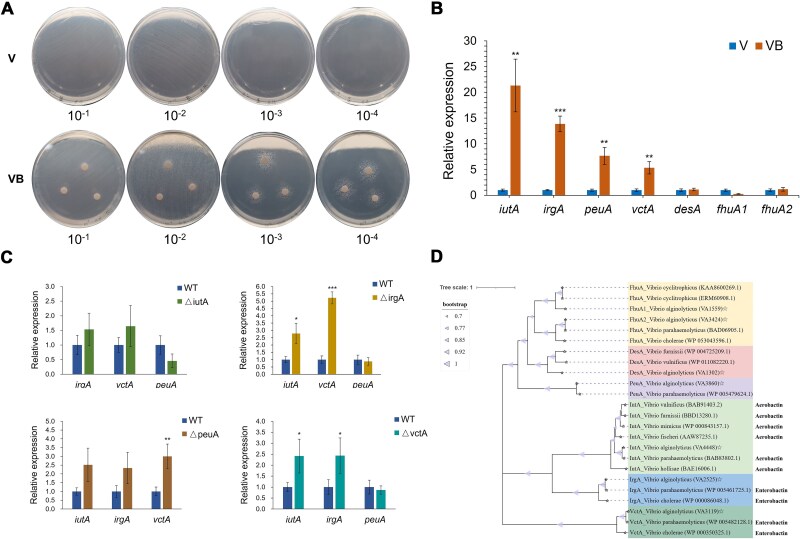
Multiple siderophore receptors mediate bacillibactin piracy in *V. alginolyticus* HN08155. (A) Growth of *V. alginolyticus* at serial 10-fold dilutions on iron-limited agar plates with or without bacillibactin supplementation. V: *V. alginolyticus* monoculture without bacillibactin. VB: *V. alginolyticus* cultured with bacillibactin supplementation. (B) qPCR analysis of relative expression levels of siderophore receptor genes in the VB group (bacillibactin-supplemented) compared to the V group (control). (C) qPCR validation of siderophore receptor gene expression in mutant strains lacking specific receptor genes, highlighting functional overlap. (D) Maximum-likelihood phylogenetic tree of *V. alginolyticus* siderophore receptors (hollow pentagram) and functionally characterized homologs. NCBI protein accession numbers are provided.

Growth assays on iron-limited agar revealed a striking bacillibactin-dependent colony phenotypic shift in *V. alginolyticus* HN08155. At 10−100× dilutions, high-density microcolonies with reduced dimensions formed, indicative of iron-restricted growth, while nearly complete growth arrest occurred at 10^3^−10^4^× dilutions. Remarkably, exogenous bacillibactin supplementation rescued growth: colonies expanded at 10−100× dilutions, with enhanced peri-disk proliferation. At higher dilutions (10^3^−10^4^×), robust colony formation was restricted to bacillibactin-proximal zones (Fig. 4A), revealing a clear distance-dependent growth gradient due to limited siderophore diffusion [31].

Genomic analysis of *V. alginolyticus* HN08155 revealed seven putative siderophore receptor homologs, yet their functional roles in heterologous bacillibactin acquisition required elucidation. To address this, we examined the expression profiles of these receptor genes under iron-limited conditions with or without bacillibactin supplementation. Integrated qPCR and transcriptomic analyses demonstrated that bacillibactin specifically triggered significant upregulation (*P* < .01) of four receptor genes—*iutA*, *irgA*, *peuA*, and *vctA*, with *iutA* and *irgA* exhibiting the highest fold changes (Fig. 4B, Supplementary Fig. S7A). To dissect functional contributions, we generated knockout mutants of these four receptors (Supplementary Fig. S7B). Growth assays under iron-limited conditions revealed that single-gene deletions did not completely abrogate bacillibactin utilization, suggesting functional overlap. However, the Δ*irgA* mutant displayed significantly impaired growth compared to wild type (WT) on bacillibactin-supplemented agar, while Δ*iutA*, Δ*peuA*, and Δ*vctA* mutants showed no pronounced growth defects (Supplementary Fig. S7C).

Further analysis of receptor gene expression in the mutants revealed distinct compensatory upregulation patterns. In the Δ*iutA* mutant, transcript levels of *irgA* and *vctA* were elevated. The Δ*irgA* mutant displayed significant upregulation of *iutA* and *vctA* (*P* < .05). Similarly, the Δ*peuA* mutant showed upregulated *irgA*, *iutA*, and *vctA*, with a significant increase in *vctA* expression (*P* < .01). In the Δ*vctA* mutant, *irgA* and *iutA* were significantly upregulated (*P* < .05) (Fig. 4C). These findings indicate functional overlap among IrgA, IutA, and VctA in heterologous bacillibactin acquisition, as deletion of one receptor triggers compensatory upregulation of others, thereby maintaining siderophore scavenging capacity. This functionally overlapping likely explains why single receptor knockouts failed to abolish bacillibactin scavenging (Supplementary Fig. S7C). Phylogenetic analysis further corroborates this compensatory mechanism, showing that IutA, IrgA, and VctA cluster within a clade of characterized siderophore (aerobactin and enterobactin) receptors (Fig. 4D). Such functional overlap in the receptor repertoire may enhance the competitive fitness of *V. alginolyticus* HN08155 in iron-limited niches by enabling efficient exploitation of bacillibactin and structurally analogous siderophores.

### Bacillibactin triggers global transcriptional changes to empower *V. alginolyticus* HN08155 fitness under iron limitation

To delineate the mechanistic basis of bacillibactin-mediated fitness in *V. alginolyticus* HN08155 under iron-limited conditions, we performed transcriptomic profiling through RNA sequencing. Principal component analysis (PCA) and correlation matrix analysis revealed clear segregation between bacillibactin-supplemented (VB) and control (V) groups (Supplementary Fig. S8A and B), demonstrating substantial transcriptional changes. Global analysis identified 2601 differentially expressed genes (DEGs), representing 49.0% of the annotated genome, with 1363 genes upregulated and 1238 downregulated (Supplementary Fig. S8C). Notably, 2105 genes (39.7% of the genome) exhibited conserved upregulation patterns in the VB group through hierarchical clustering analysis (Supplementary Fig. S8D).

**Figure 5:**
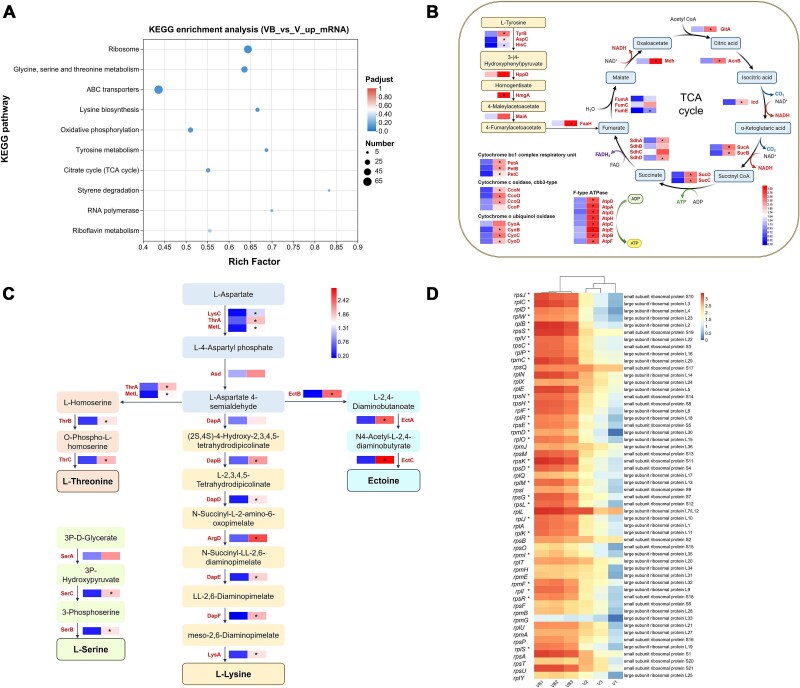
Transcriptomic effects of bacillibactin supplementation on *V. alginolyticus* HN08155 under iron-limited conditions. (A) KEGG pathway enrichment analysis of DEGs between the bacillibactin-supplemented (VB) and control (V) groups under iron-limited conditions. (B) Differential expression profiles of energy metabolism-related genes, including the TCA cycle, L-tyrosine degradation, and electron transport chain components. (C) Differential expression analysis of genes involved in amino acid (and derivative) biosynthesis, including pathways for L-lysine, L-threonine, L-serine, and ectoine. (D) Differential expression analysis of ribosome-related genes. Asterisks denote genes with |log_2_(fold change)| ≥ 1.0 and adjusted *P*-value <.05.

KEGG pathway enrichment analysis revealed that bacillibactin triggers distinct metabolic adjustment, with seven pathways showing statistically significant activation (*P*adjust < .05) in the VB group: ribosome, glycine, serine and threonine metabolism, ATP-Binding Cassette (ABC) transporters, lysine biosynthesis, oxidative phosphorylation, tyrosine metabolism, and TCA cycle (Fig. 5A). Notably, the analysis did not reveal any significantly downregulated pathways (*P*adjust >.05), indicating that bacillibactin primarily drives metabolic activation rather than suppression. GO enrichment analysis further demonstrated significant upregulation of biological processes related to amino acid/peptide metabolism and protein biosynthesis in the VB group (Supplementary Fig. S8E).

More specifically, the majority of genes involved in the TCA cycle were significantly upregulated in the VB group, mirroring growth-enhanced phenotypes where TCA activation fuels heightened energy demands. Parallel activation of L-tyrosine degradation genes facilitated fumarate accumulation and its integration into the TCA cycle (Fig. 5B), establishing a metabolic loop to sustain energy production. This was further amplified by significant upregulation of electron transport chain components, including cytochrome bc1 complex respiratory unit, cbb3-type cytochrome c oxidase, cytochrome o ubiquinol oxidase, and F-type ATP synthase (Fig. 5B), collectively enhancing respiratory efficiency and ATP generation. A metabolic shift toward amino acid production was further evidenced by pronounced upregulation of genes involved in the biosynthesis of L-threonine, L-serine, L-lysine, and ectoine (Fig. 5C). Significantly, most of the ribosomal pathway genes exhibited pronounced upregulation in the VB group (Fig. 5D), highlighting a bacillibactin-mediated adaptive strategy to enhance protein synthesis capacity under iron limitation. Together, these findings underscore bacillibactin’s central role in metabolic remodeling, enabling *V. alginolyticus* HN08155 to counteract iron scarcity by synergistically integrating protein synthesis with energy production, thereby ensuring a competitive fitness advantage in iron-limited environments.

While our experimental data demonstrate that endogenous vibrioferrin production in *V. alginolyticus* HN08155 remains insufficient to counteract severe iron limitation (Fig. 1D), transcriptomic profiling revealed striking activation of the entire vibrioferrin biosynthesis and transport operon in the VB group (Supplementary Fig. S9A). We propose that bacillibactin supplementation provides essential metabolic priming, redirecting cellular resources toward enhanced endogenous siderophore biosynthesis. This cross-feeding mechanism likely initiates a self-sustaining iron acquisition cascade, whereby vibrioferrin-mediated iron retrieval compensates for the diminishing bioavailability of bacillibactin under prolonged iron restriction. Moreover, significant upregulation of two siderophore importer gene clusters, *vctPDGC* and *fhuCDB*, was observed in the VB group (Supplementary Fig. S9B). These clusters are implicated in the transport of siderophores enterobactin and aerobactin [32, 33], respectively, suggesting their potential role in mediating the intracellular transport of bacillibactin–iron complexes across the inner membrane.

### *Vibri alginolyticus* HN08155 acquires growth advantage in iron-limited coculture with siderophore-producing *B. velezensis*

**Figure 6:**
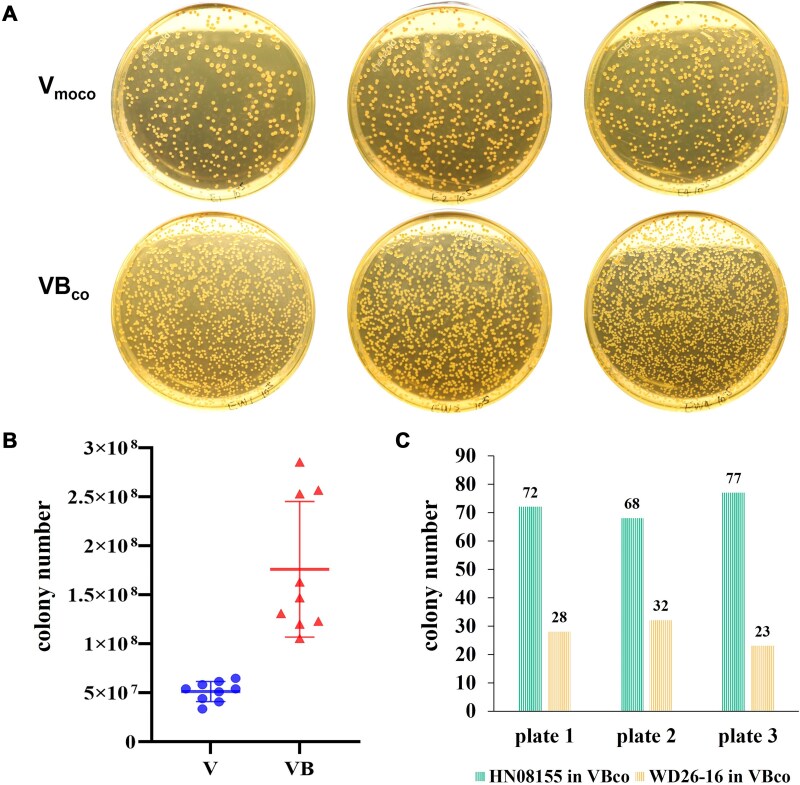
Competitive interactions between V. *a*lginolyticus HN08155 and B. *c*elezensis WD26-16 under iron-limited conditions. (A) Morphological comparison of V. *a*lginolyticus HN08155 colonies on TCBS agar after 24 h of monoculture in iron-limited medium (Vmoco) versus coculture with B. *v*elezensis WD26-16 in iron-limited coculture medium (VBco). Yellow colonies indicate V. *a*lginolyticus, while B. *v*elezensis does not grow on TCBS agar. (B) Quantification of V. *a*lginolyticus CFUs in monoculture (V) versus co-culture (VB) under iron-limited conditions. (C) Proportional CFU counts of V. *a*lginolyticus and B. *v*elezensis in coculture, highlighting the dominance of V. *a*lginolyticus under iron-limited conditions.

To investigate whether the siderophore bacillibactin produced by *B. velezensis* fuels pathogenic *V. alginolyticus* proliferation through siderophore piracy, we performed coculture experiments in iron-limited medium with a 1:1 inoculation ratio. Strikingly, liquid cocultures of *V. alginolyticus* HN08155 and *B. velezensis* WD26-16, when plated on selective TCBS agar that supports the growth of HN08155 but not WD26-16, resulted in significantly denser colonies compared to HN08155 monocultures (Fig. 6A). Quantitative CFU analysis further revealed a 3.4-fold increase in the growth of strain HN08155, from 5.14 × 10^7^ CFU/ml in monoculture to 1.76 × 10^8^ CFU/ml in coculture (Fig. 6B), demonstrating that bacillibactin-mediated iron acquisition facilitates *V. alginolyticus* proliferation.

To further evaluate interspecies competition under iron restriction, we quantified the relative abundances of *V. alginolyticus* HN08155 and *B. velezensis* WD26-16 after 24 h of coculture using colony-specific PCR. Across three independent replicates, strain HN08155 accounted for 72, 68, and 77 colonies, respectively, out of 100 randomly selected colonies per plate (Fig. 6C), yielding a relative fitness constant of 2.6 relative to strain WD26-16. The pronounced growth disparity underscores the competitive advantage of *V. alginolyticus* in iron-limited medium, primarily attributed to its ability to exploit siderophores produced by *B. velezensis*, thereby enhancing its proliferation and ecological dominance.

## Discussion

*Vibrio alginolyticus* strains are notably rich in siderophore receptors, enabling them to efficiently “cheat” by utilizing siderophores produced by other species [9]. However, the identity of microbial “providers” that unwittingly supply siderophores to *V. alginolyticus* remains poorly characterized. Our supernatant feeding assay revealed that about 17.0% of siderophore-producing isolates from mariculture systems enhanced the growth of pathogenic *V. alginolyticus* HN08155 under iron-limited conditions. These promotive siderophore producers primarily belong to *Vibrio*, *Bacillus*, and *Shewanella* (Fig. 1C), which are ubiquitously associated with aquaculture microbiomes [34]. Notably, strain HN08155 demonstrated limited competitiveness in iron acquisition via its native siderophore (Fig. 1D), vibrioferrin, which exhibits weaker iron-binding affinity (logβ_110_ = 24.02) compared to other marine siderophores [35]. This inherent deficiency may compel *V. alginolyticus* to adopt a parasitic iron acquisition strategy, exploiting high-affinity xenosiderophores secreted by neighboring microbes like *Vibrio* or *Bacillus* species within polymicrobial niches. Such ecological cheating behavior is observed in *V. cholerae*, which hijacks the high-affinity siderophore enterobactin from *E. coli* to overcome environmental and host iron restrictions [12].

Our results reveal an ecological interaction wherein the marine pathogen *V. alginolyticus* exploits siderophores produced by the probiotic *B. velezensis* to enhance its proliferation. This cross-genus interaction is mediated by bacillibactin, a catechol-type siderophore commonly synthesized by *Bacillus*, particularly plant-associated species (e.g. *B. subtilis*, *B. velezensis*, and *B. amyloliquefaciens*) for iron acquisition [28]. While bacillibactin is well documented as an antimicrobial agent against fungal phytopathogens and bacterial pathogens like *Staphylococcus aureus*, *Enterococcus faecalis*, *Pseudomonas aeruginosa*, and *Klebsiella pneumoniae* [36–38], we present evidence of its contrasting role as a nutritional resource for marine *Vibrio* pathogens in aquatic ecosystems. This evolutionary divergence is further highlighted by recent evidence demonstrating that *B. subtilis* suppresses colony spreading and secondary metabolite production (e.g. pyoverdine) in *P. marginalis* through bacillibactin-mediated iron competition—a conserved antagonistic mechanism across *B. subtilis* and multiple *Pseudomonas* species [39]. Strikingly, our work demonstrates that *V. alginolyticus* subverts this system, co-opting bacillibactin produced by *B. velezensis* for iron acquisition and proliferation, contrasting with its typically reported antagonistic roles in terrestrial *Bacillus*–pathogen interactions [39, 40].

Comparative genomic analysis reveals a remarkable evolutionary conservation of bacillibactin BGCs across marine *Bacillus* species. Notably, 86.1% of the surveyed genomes retain intact BGCs that exhibit high amino acid identity (Fig. 3). This pervasive conservation pattern strongly suggests that bacillibactin-mediated iron acquisition represents not merely an evolutionary relic but rather a crucial functional adaptation that has been selectively maintained within marine *Bacillus* populations. Such conservation contrasts with the frequent loss of siderophore BGCs observed in marine *Vibrio* species, where ~50% of strains lack autonomous siderophore BGCs but retain an extensive repertoire of siderophore receptor systems [8, 9]. Remarkably, we demonstrate that the contrasting iron acquisition strategies between *Bacillus* and *Vibrio* converge on catecholate-mediated systems, with *Bacillus* synthesizing bacillibactin as a public good and *Vibrio* exploiting this siderophore through conserved receptors. This duality underscores the evolutionary interplay between cooperative synthesis and exploitative competition to sustain microbial coexistence [41].

It is noteworthy that all marine *B. velezensis* isolates harboring bacillibactin BGCs exhibit over 97% sequence identity with the bacillibactin-producing strain WD26-16 (Fig. 3), indicating their genetic potential to synthesize the identical siderophore bacillibactin. This exceptionally high conservation of bacillibactin BGCs among *B. velezensis* strains, coupled with its well-documented probiotic nature in marine environments—including enhancing digestion, promoting nutrient absorption, and modulating the immunity of marine hosts—raises critical concerns regarding the widespread use of *B. velezensis* as a probiotic in aquaculture systems [42–44]. This concern is particularly salient given the observed growth-promoting effects of WD26-16-derived bacillibactin on *V. alginolyticus*. Specifically, bacillibactin-producing strains may inadvertently enhance the environmental resilience of *V. alginolyticus*—a pathogen linked to vibriosis outbreaks [45, 46]—by facilitating iron acquisition through siderophore-mediated competition. Such interaction mirrors an evolutionarily entrenched symbiosis, akin to rhizosphere *B. velezensis* species stimulating resident rhizosphere *P. stutzeri* for plant health through metabolic interactions [47]. In marine aquaculture microbiomes, however, this dynamic may favor pathogen persistence, exacerbating disease transmission risks. Our findings highlight the potential unintended consequences of introducing siderophore-proficient probiotics into such ecosystems.

Central to efficient bacillibactin piracy in *V. alginolyticus* is its versatile arsenal of functionally overlapping siderophore receptors (IutA, IrgA, VctA). Our discovery of compensatory upregulation of alternative receptors in knockout mutants (e.g. *iutA* and *vctA* in Δ*irgA*) unveils a fail-safe mechanism essential for survival in iron-limited niches. Phylogenetic clustering of IutA, IrgA, and VctA with characterized siderophore receptors further supports evolutionary conservation of this scavenging mechanism. Notably, homologs of VctA and IrgA mediate enterobactin acquisition in *V. cholerae* and *V. parahaemolyticus* [32, 48], while IutA facilitates aerobactin utilization in *V. parahaemolyticus* [49], suggesting functional convergence enabling cross-reactivity with bacillibactin. Catechol-type siderophores, particularly enterobactin and bacillibactin, are recognized for their strong affinity for iron [50]. In *V. alginolyticus*, the abundance of siderophore receptors may confer a competitive advantage against microbial rivals by broadening the spectrum of high-affinity xenosiderophores, a critical advantage in marine ecosystems and host–pathogen interactions where *Vibrio* species vie for scarce iron resources.

Transcriptomic profiling uncovers a bacillibactin-fueled metabolic network in *V. alginolyticus*, where siderophore piracy facilitates survival under iron-restricted conditions by orchestrating the upregulation of energy metabolism and protein synthesis pathways. Notably, the pronounced upregulation of the TCA cycle and oxidative phosphorylation aligns with prior reports linking siderophore-mediated iron acquisition to enhanced respiratory efficiency in *P. aeruginosa* and *E. coli* under iron scarcity [51, 52]. Concurrent induction of amino acid biosynthesis pathways (e.g. serine, threonine, lysine) and ribosomal machinery underscores a resource allocation strategy favoring rapid biomass accumulation. This parallels observations in *V. parahaemolyticus*, where iron scavenging is tightly coupled to the activation of anabolic pathways to support virulence and proliferation [53].

Our co-culture experiments further demonstrate that *V. alginolyticus* hijacks bacillibactin to outcompete its producer, *B. velezensis*, in iron-limited environments. This interaction exemplifies the “public goods dilemma” in microbial communities, where nonproducers benefit from siderophores synthesized by cooperative neighbors [54]. Our study advances the current understanding by demonstrating that pathogenic *V. alginolyticus* exploits heterologous siderophore uptake to establish niche dominance, a strategy with direct implications for its persistence in mariculture systems where iron availability dictates microbial competition [5].

In summary, the present study reveals an ecological interaction wherein the probiotic *B. velezensis* inadvertently fuels the proliferation of the opportunistic pathogen *V. alginolyticus* through cross-genus siderophore piracy. This finding underscores the context-dependent nature of the ecological interactions between *B. velezensis* and other microorganisms, highlighting the dual role of bacillibactin as both a competitive weapon and a cooperative public good within microbial communities. Moreover, this study broadens our understanding of the interactions between two of the most extensively studied bacterial genera in marine aquaculture. Future research should explore the prevalence of xenosiderophore piracy across marine *Vibrio*–*Bacillus* systems and investigate the receptor–specificity dynamics that modulate siderophore piracy efficacy.

## Supplementary Material

Supplementary_Figures_ycaf132

Supplementary_Tables_ycaf132

## Data Availability

Raw RNA sequencing data generated in this work have been deposited in the NCBI Sequence Read Archive (SRA) (http://www.ncbi.nlm.nih.gov/sra) under BioProject accession number PRJNA1244722. All mutant strains generated in this study are available from the corresponding author upon request.
