# Supplementary material for: Probiotic paradox: bacillibactin from Bacillus velezensis drives pathogenic Vibrio alginolyticus proliferation through siderophore piracy
Source: ISME Commun. 2025 Aug 1;5(1):ycaf132. doi: 10.1093/ismeco/ycaf132 (PMC12366790; doi:10.1093/ismeco/ycaf132)
Supplement: Supplementary_Figures_ycaf132 [file supplementary_figures_ycaf132.pdf]

Supplementary Figures for:

Probiotic paradox: Bacillibactin from *Bacillus velezensis* drives pathogenic *Vibrio alginolyticus* proliferation through siderophore piracy

Yanhua Zeng[1, 2, 3], Haimin Chen[1, 2], Xiaoxiao Gong[1, 2], Manwei Jiang[1, 2], Ni Liu[1, 2], Wen Li[1, 2], Na Zhang[1, 2], Hao Long[1, 2, 3], Aiyou Huang[1, 2], Zhenyu Xie[1, 2, 3 *]

[1] Key Laboratory of Tropical Hydrobiology and Biotechnology of Hainan Province, School of Marine Biology and Fisheries, Hainan University, Haikou, Hainan 570228, PR China

[2] Wenchang Advanced Fisheries Research Institute, Hainan University, Wenchang, Hainan 571300, PR China

[3] State Key Laboratory of Marine Resource Utilization in South China Sea, Hainan University, Haikou, Hainan 570228, PR China

Address of corresponding author: Zhenyu Xie

State Key Laboratory of Marine Resource Utilization in South China Sea, Hainan University. 58 Renmin Avenue, Haikou, Hainan 570228, PR China.

Email: xiezyscuta@163.com

Running title: Siderophore-mediated interaction between *Bacillus* and *Vibrio*

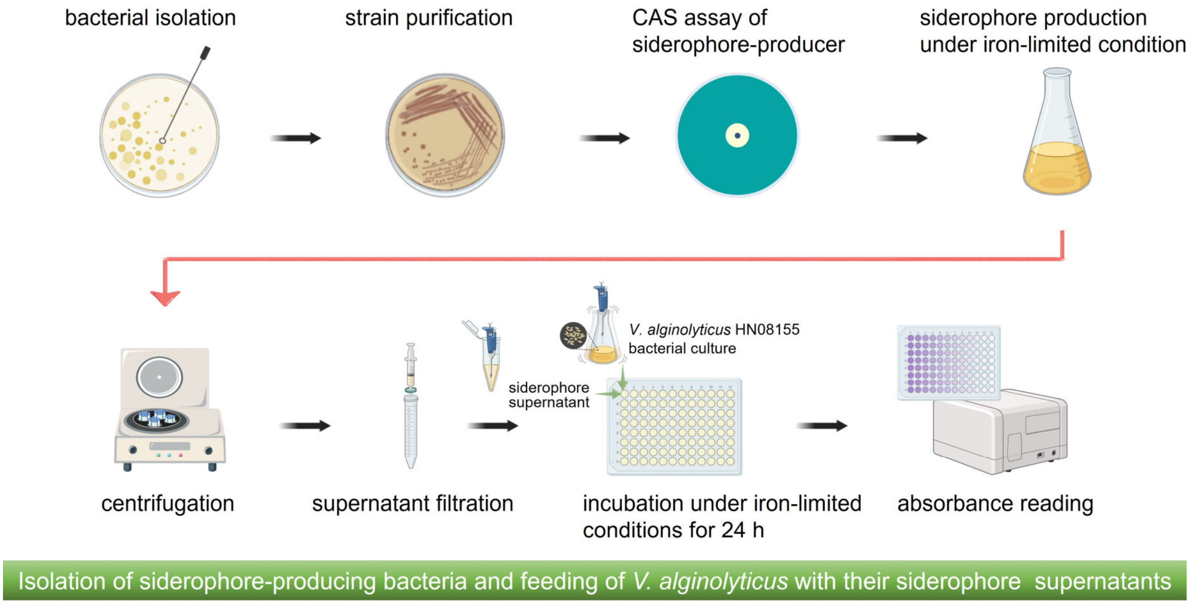

Supplementary **Fig. S1** Schematic workflow for isolating siderophore-producing bacteria and conducting supernatant experiments.

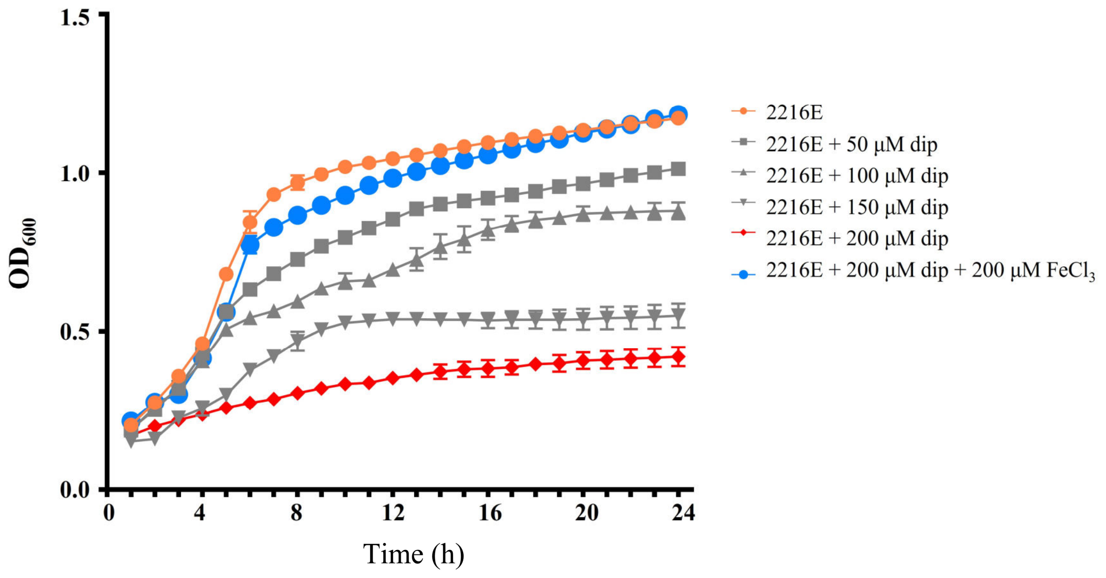

Supplementary **Fig. S2** Growth curves of *V. alginolyticus* HN08155 cultured in 2216 medium supplemented with varying concentrations of the iron chelator 2,2′-bipyridine.

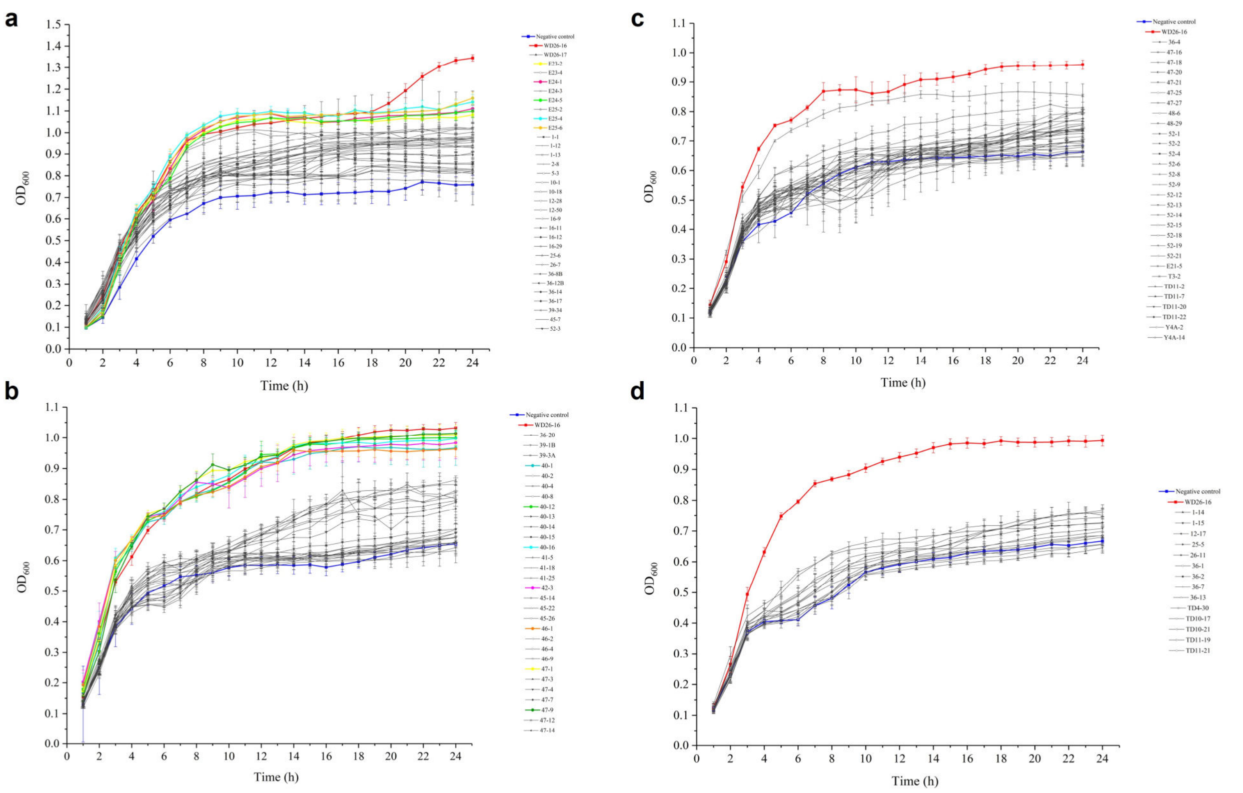

Supplementary **Fig. S3** Growth curves of *V. alginolyticus* HN08155 cultured in iron-limited media supplemented with siderophore-enriched supernatants from different siderophore-producing bacterial strains. The blue curve represents the negative control without siderophore supplementation, while all other colored curves represent strains demonstrating statistically significant growth enhancement relative to the control.

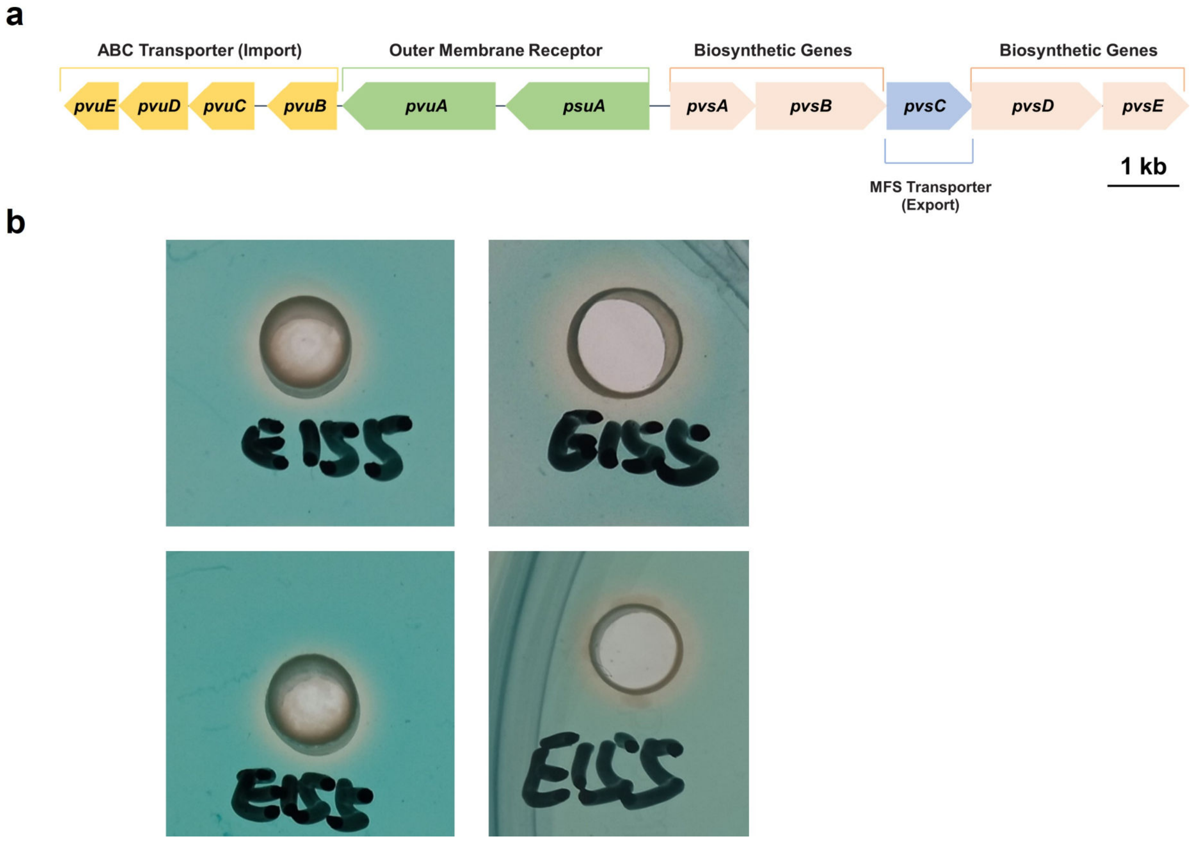

Supplementary **Fig. S4** The siderophore biosynthetic gene cluster and siderophore detection in *V. alginolyticus* HN08155. **a** Genomic organization of the siderophore biosynthesis gene cluster in strain HN08155. **b** CAS plate assay showing low levels of siderophore production by strain HN08155 (representative replicates).

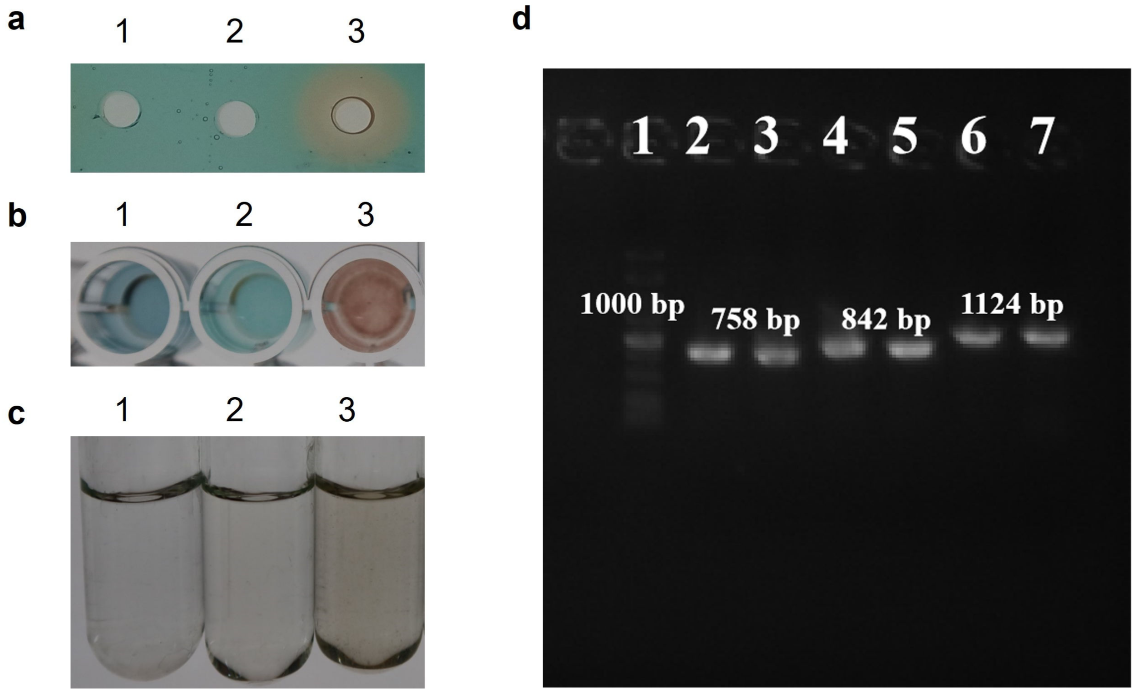

Supplementary **Fig. S5** Detection of siderophores in crude extracts of *B. velezensis* WD26-16 and amplification of the corresponding biosynthetic genes. **a** CAS plate assay for crude siderophores. **b** CAS liquid assay for crude siderophores. **c** Ferric perchlorate assay for hydroxamate-type siderophore. In panels a–c, treatments: 1, sterile water; 2, iron-limited medium; 3, crude siderophores from strain WD26-16. **d** Electrophoresis of catechol-type siderophore biosynthesis genes: *dhbA* (lanes 2–3), *dhbB* (lanes 4–5), *dhbC* (lanes 6–7).

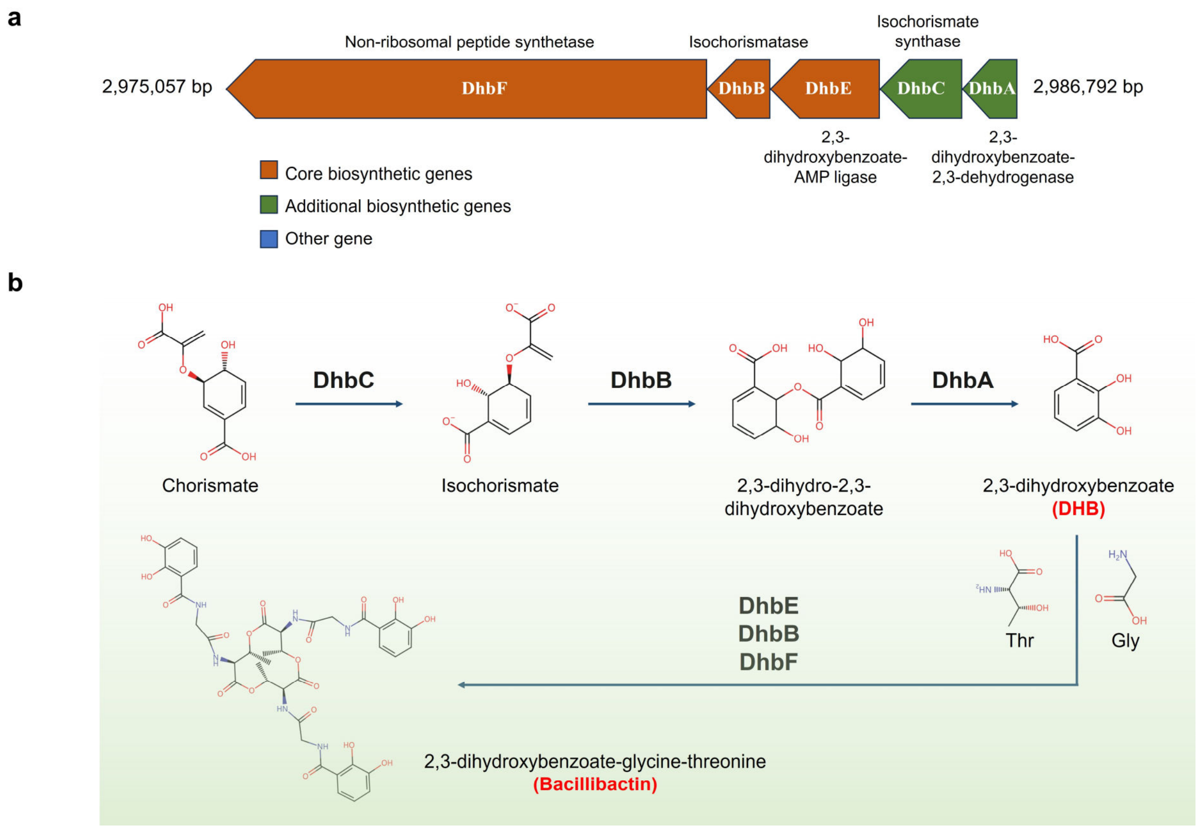

Supplementary **Fig. S6** Genomic organization of the bacillibactin BGC and biosynthetic pathway in *B. velezensis* WD26-16. **a** Genomic organization of the bacillibactin BGC in strain WD26-16, annotated using antiSMASH. **b** Proposed biosynthetic pathway for bacillibactin in strain WD26-16, reconstructed through comparative genomic analysis.

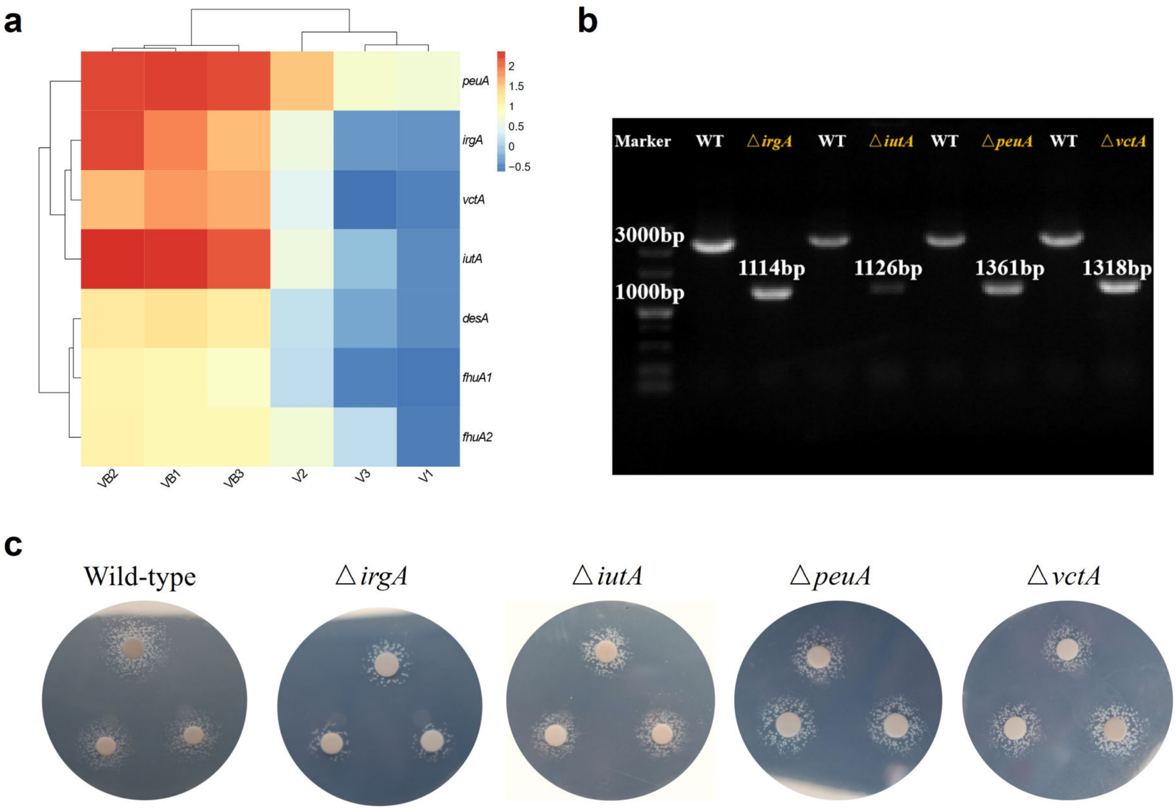

Supplementary **Fig. S7** Functional analysis of siderophore receptor genes in *V. alginolyticus* HN08155 during bacillibactin utilization. **a** Transcriptomic analysis of relative expression levels of siderophore receptor genes in the VB group (bacillibactin-supplemented) compared to the V group (control). **b** Electrophoretic validation of the siderophore receptor gene knockout mutant. PCR amplification of the wild-type (WT) strain yielded a full-length target gene fragment, whereas all four knockout mutants produced truncated fragments confirming successful gene deletion. **c** Growth phenotype of the WT and mutants under iron-limited conditions supplemented with bacillibactin.

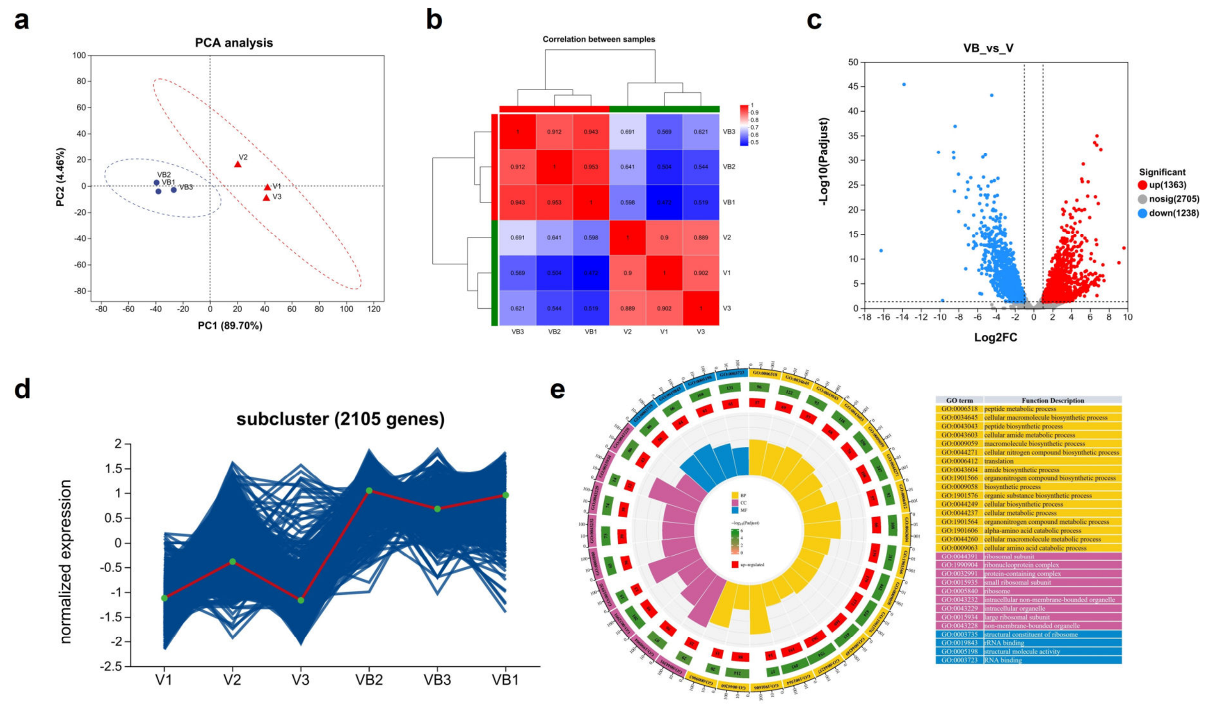

Supplementary **Fig. S8** Comparative transcriptomic analysis of *V. alginolyticus* HN08155 cultures under iron-limited conditions with bacillibactin supplementation (VB) versus control (V). **a** Principal component analysis (PCA) of global gene expression profiles, demonstrating distinct clustering patterns between VB and V groups. **b** Hierarchical clustering heatmap of inter-sample Pearson correlation coefficients, revealing transcriptional divergence modulated by bacillibactin availability. **c** Volcano plot of DEGs with thresholds set at $|\log_2(\text{fold change})| \geq 1$ and adjusted p-value < 0.05. Red points denote 1363 bacillibactin-induced upregulated genes, while blue points represent 1238 downregulated genes. **d** Hierarchical subcluster analysis of DEGs identifying co-regulated gene modules. **e** GO enrichment analysis showing the top 30 most significantly enriched GO terms for up-regulated DEGs between VB and V groups.

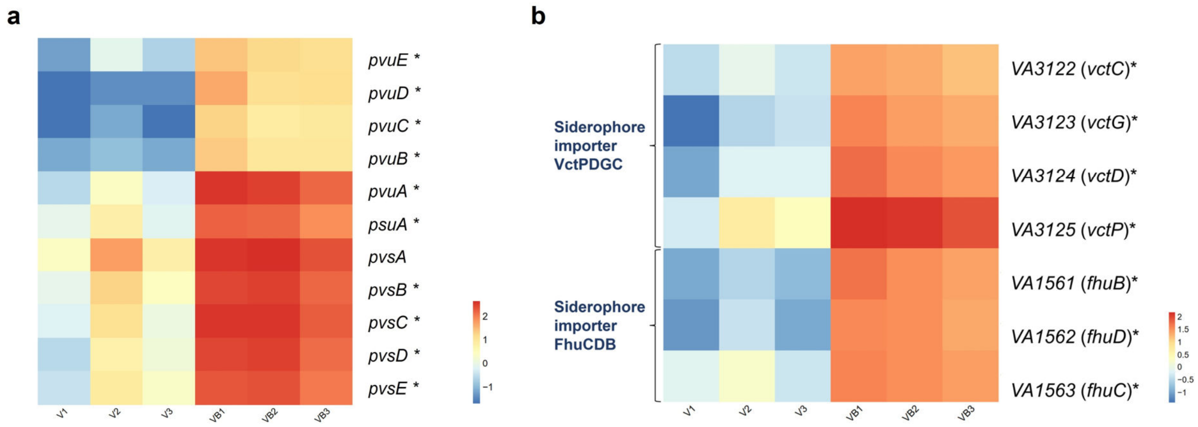

Supplementary **Fig. S9** Influence of bacillibactin on the expression of siderophore biosynthesis and importer genes in *V. alginolyticus* HN08155. **a** Heatmap visualization of transcriptome data depicting expression differences in siderophore biosynthesis genes between bacillibactin-supplemented (VB) and control (V) groups. **b** Heatmap analysis of differentially expressed siderophore importer genes in VB versus V groups. Asterisks (*) indicate genes with significant differential expression.
